# Association between chronic obstructive pulmonary disease and serum lipid levels: a meta-analysis

**DOI:** 10.1186/s12944-018-0904-4

**Published:** 2018-11-21

**Authors:** Lingling Xuan, Feifei Han, Lili Gong, Yali Lv, Zirui Wan, He Liu, Dongsu Zhang, Yangjie Jia, Song Yang, Lulu Ren, Lihong Liu

**Affiliations:** Department of Pharmacy, Beijing Chao-Yang Hospital, Capital Medical University, Beijing, China

**Keywords:** COPD, dyslipidemia, high-density lipoprotein cholesterol, low-density lipoprotein cholesterol, total cholesterol, triglyceride

## Abstract

**Background:**

Metabolic syndrome is a common extrapulmonary comorbidity in patients with chronic obstructive pulmonary disease (COPD). However, the reported relationship of COPD with dyslipidemia, an important component of metabolic syndrome, is ambiguous. The aim of this meta-analysis is to investigate the association between COPD and the serum levels of high-density lipoprotein cholesterol (HDL), low-density lipoprotein cholesterol (LDL), total cholesterol (TC), and triglyceride (TG).

**Methods:**

The PubMed and Embase databases were searched to find potential studies using the search terms of (“dyslipidemia” or “HDL” or “LDL” or “cholesterol” or “triglyceride”) and COPD. We also performed subgroup analysis enrolling patients who were not receiving treatment for dyslipidemia. Mean differences (MD) with 95% confidence intervals (CI) were estimated with random effects models.

**Results:**

A total of 11 studies comprising 615 cases and 471 controls were included in the study. No significant differences were found in the HDL (MD = −2.55, 95% CI [−6.03, 0.93], P = 0.15), LDL (MD = −2.25, 95% CI [−13.36, 8.86], P = 0.69), TC (MD = −2.69, 95% CI [−13.30, 7.92], P = 0.62), and TG (MD = 6.90, 95% CI [−2.81, 16.60], P = 0.16) levels of the 2 groups. However, subgroup analysis enrolling patients who were not receiving treatment for dyslipidemia showed that TG levels were higher in patients with stable COPD than in healthy individuals (MD = 16.35, 95% CI [5.90, 26.80], P = 0.002).

**Conclusions:**

Excluding the impact of hypolipidemic treatment on serum lipid profile, TG levels were higher in patients with COPD than in healthy individuals. This meta-analysis suggested that physicians should screen COPD patients for elevated TG levels to reduce the risk of cardiovascular morbidity and mortality.

## Background

Chronic obstructive pulmonary disease (COPD), a progressive disease of the lungs characterized by persistent airflow limitation, is one of the main causes of morbidity and mortality worldwide. Epidemiological studies revealed that COPD patients have various extrapulmonary comorbidities such as coronary heart disease (CHD), metabolic syndrome, and depression [1]. Extrapulmonary comorbidities increase the risk of hospitalisation and mortality in COPD patients. An increasing number of COPD patients die from systemic comorbidities rather than respiratory failure [2, 3].

CHD, also known as atherosclerotic heart disease and coronary artery disease, is common among patients with COPD. A strong association between CHD and COPD has been widely evaluated. COPD is an independent risk factor for CHD and, conversely, CHD is associated with the diagnosis and severity of COPD [4]. For example, in a population-based survey, CHD was reported in 13% of patients diagnosed with COPD and 4% in subjects with normal spirometry [5]. Another study showed that, the prevalence of CHD among adults aged 45 years and older who had COPD was 12.7% [6]. Although it varies in some studies, the reported prevalence of CHD in subjects with COPD can be up to 60% [7, 8].

Metabolic syndrome is another common comorbidity of COPD. The prevalence of metabolic syndrome has been reported to be 20% to 50% in people with COPD [9, 10]. Previously conducted studies reported an increased prevalence of metabolic syndrome in COPD patients compared to healthy subjects. How people with COPD develop metabolic syndrome is still unclear, but researchers have postulated that cigarette smoking, systemic inflammation and physical inactivity may play a role in the development of metabolic syndrome in COPD patients [11, 12].

Dyslipidemia, a major risk factor for CHD and metabolic syndrome, is characterized by a cluster of lipid abnormalities such as an elevated level of triglyceride (TG), a reduced level of high-density lipoprotein cholesterol (HDL) and an increased level of low-density lipoprotein cholesterol (LDL). A number of studies have evaluated the relationship between COPD and blood lipid profiles with inconsistent results. While some authors reported reduced serum levels of HDL or increased serum levels of TG in COPD patients [13, 14], others did not observe any significant changes in lipid serum profiles [15]. The objective of the present study was to investigate the association between COPD and the serum levels of HDL, LDL, total cholesterol (TC), and TG.

## Methods

### Search strategy

We searched PubMed and Embase for related studies published from database inception to April 2018, and the language was restricted to English. The search was performed using the search terms of (“dyslipidemia” or “HDL” or “LDL” or “cholesterol” or “triglyceride”) and COPD. We also screened the reference lists of included studies to identify any potentially relevant studies.

### Inclusion criteria

The inclusion criteria were as follows:

1. There should be at least 2 groups (COPD group and healthy control group);
2. The COPD patients were in stable period;
3. The study should report the serum levels of HDL, or/and LDL, or/and TC, or/and TG of these 2 groups.

### Exclusion criteria

The studies met with the following items would be excluded:

1. Reviews, commentaries, personal communications, proceedings, and case observations;
2. Animal studies or *in vitro* studies;
3. Significant different between the 2 groups for baseline age, gender, or body mass index (BMI);
4. Use of systemic corticosteroid in the preceding three months;
5. Respiratory diseases other than COPD.

### Data extraction

The following information was extracted from each included study: first author, the year of publication, location, sample size, age range, and BMI. Outcomes extracted included HDL, LDL, TC, and TG levels. Two investigators independently extracted data from the selected studies based on the predetermined inclusion and exclusion criteria. Any disagreements were solved with the help of a third reviewer, when necessary.

### Statistical analysis

Statistical analysis was performed using Review Manager version 5.3 (Cochrane Collaboration, Baltimore, Maryland). Results were expressed as mean differences (MD) with 95% confidence intervals (CI). Random-effects model was applied in all the analysis. Cochran’s Q test and the inconsistency index (I^2^) were used to evaluate heterogeneity across the included studies. Data with P ≥ 0.10 and I^2^ ≤ 50% were defined as low heterogeneity. We assessed potential publication bias by funnel plot and Egger’s test using Stata 10.0. Sensitivity analysis was performed by sequential removal (statistics of study removed) of individual studies.

## Results

### Study selection

**Fig. 1:**
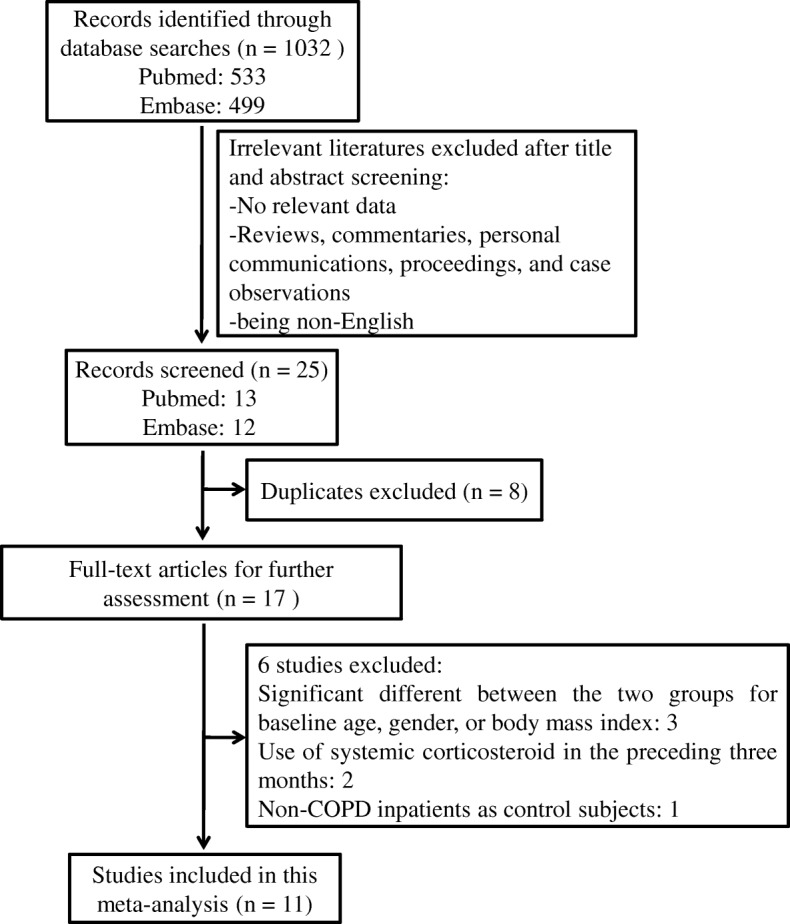
Flow diagram of study selection

Eleven eligible studies were identified. Study selection is summarized in Fig. 1. Initially, 1032 papers were identified through our search strategy. One thousand and seven studies were excluded for the following reasons: no relevant data, animal studies, reviews, commentaries, personal communications, proceedings, case observations, and being non-English. There were 17 articles remaining after duplicate results were removed. By further analyzing the full text of the 17 remaining papers, only 11 studies fulfilled all the inclusion and exclusion criteria and were included in our meta-analysis.

### Study characteristics

The characteristics of the included studies are summarized in Table 1. The publication dates of the 11 included studies ranged from 1981 to 2018 [13–23]. The included studies had been performed in Turkey, Bangladesh, China, Canada, Italy, United States, and India. A total of 1086 participants were included: 615 in the COPD group and 471 in the control group. There was no significant difference between the experimental and control groups in general information. Eleven studies reported data for HDL, 9 studies presented data for LDL and TC, and 10 studies reported data for TG.

**Table 1:** Summary of basic characteristics of selected studies for meta-analysis

Author	Year	Country	Sample size (COPD/Control)	COPD group	Control group	Age (Mean ± SD, COPD/Control)	BMI (Mean ± SD, COPD/Control)
Gunay S [13]	2016	Turkey	104/40	Stable COPD	Healthy control	64.0 ± 8.7/62.8 ± 8.9	24.72 ± 3.91/25.08 ± 2.66
Can U [14]	2015	Turkey	51/45	Stable COPD	Healthy control	56.92 ± 3.0/54.8 ± 3.8	28.76 ± 2.9/27.53 ± 3.4
Ismail M [16]	2015	Bangladesh	30/20	Stable COPD	Healthy control	NA	NA
Boyuk B [17]	2015	Turkey	43/38	Stable COPD	Healthy control	NA	26.39 ± 4.99/23.67 ± 4.75
Shen Y [18]	2013	Chinese	48/32	Stable COPD	Healthy control	62 ± 10/58 ± 11	22.57 ± 3.25/23.04 ± 2.62
Marquis K [19]	2005	Canada	38/34	Stable COPD	Healthy control	66 ± 7/63 ± 6	28 ± 5/29 ± 5
Basili S [15]	1999	Italy	90/90	Stable COPD	Healthy control	69.2 ± 8.2/67.0 ± 7.6	NA
Tisi GM [20]	1981	USA	22/22	Stable COPD	Healthy control	65.3 ± 1.9/65.3 ± 1.9	NA
Fratta Pasini AM [21]	2016	Italy	30/30	Stable COPD	Healthy control	69.3 ± 6.6/67.8 ± 6.8	28.3 ± 4.3/27.6 ± 4.8
Acharyya A [22]	2016	India	77/77	Stable COPD	Healthy control	60 ± 12/60 ± 10	23 ± 6/24 ± 4
Rafie S [23]	2018	India	82/43	Stable COPD	Healthy control	64.9 ± 7.5/63.9 ± 6.9	20.1 ± 3.9/20.7 ± 3.4

### Lipid profiles of COPD and control subjects

**Fig. 2:**
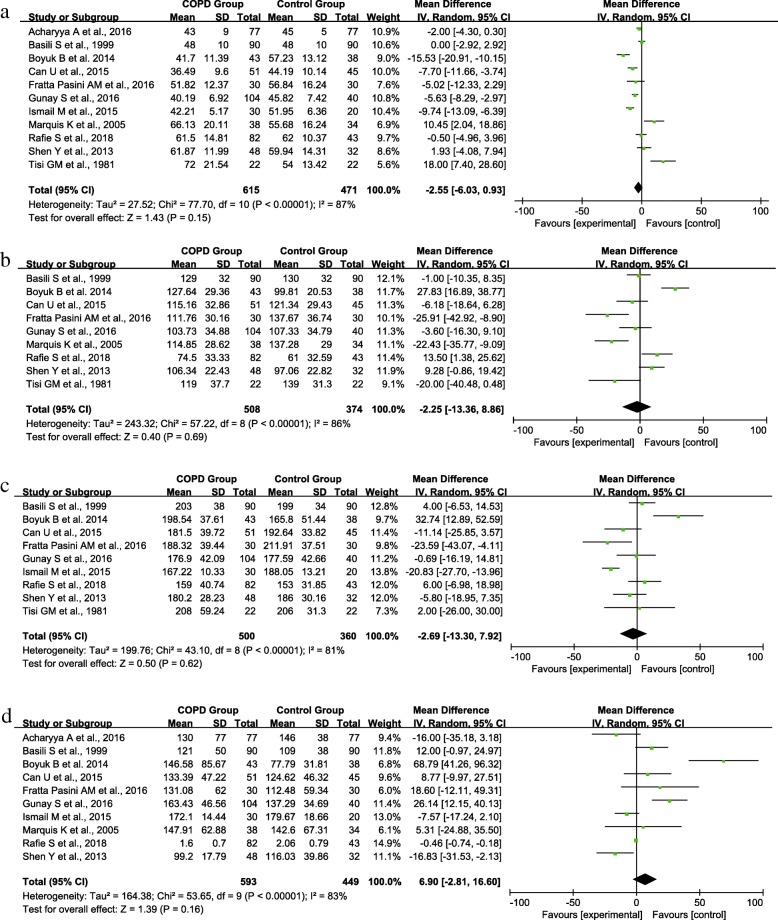
Forest plot of the (**a**) HDL, (**b**) LDL, (**c**) TC, and (**d**) TG levels comparison between the experimental and control groups

Heterogeneity of the data was observed for all the lipid profile outcomes (HDL: P < 0.001, I^2^ = 87%; LDL: P < 0.001, I^2^ = 86%; TC: P < 0.001, I^2^ = 81%; TG: P < 0.001, I^2^ = 83%). Hence, a random-effects model was used to pool the meta-analysis. No significant differences were found in the HDL (MD = −2.55, 95% CI [−6.03, 0.93], P = 0.15) (Fig. 2a), LDL (MD = −2.25, 95% CI [−13.36, 8.86], P = 0.69) (Fig. 2b), TC (MD = −2.69, 95% CI [−13.30, 7.92], P = 0.62) (Fig. 2c), and TG (MD = 6.90, 95% CI [−2.81, 16.60], P = 0.16) (Fig. 2d) levels of the 2 groups.

### Subgroup analysis

**Fig. 3:**
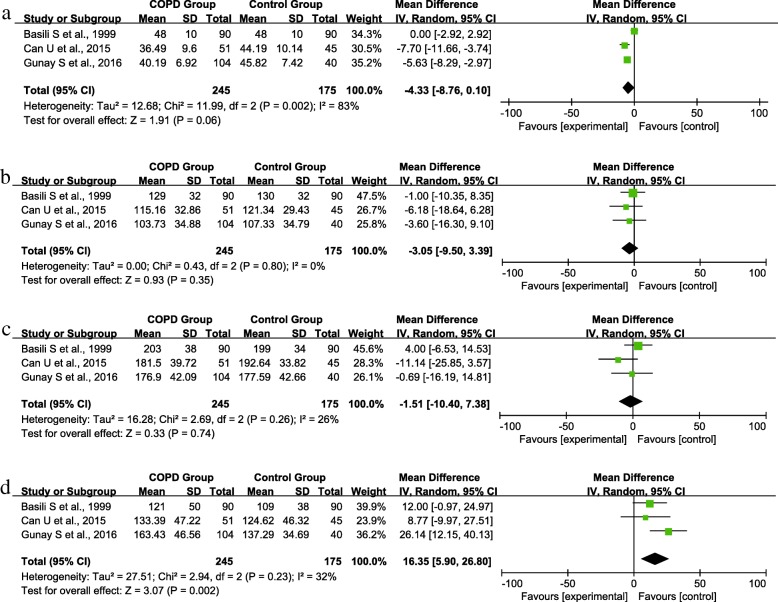
Forest plot of the (**a**) HDL, (**b**) LDL, (**c**) TC, and (**d**) TG levels comparison between the experimental and control groups in the subgroup analysis enrolling patients who were not receiving treatment for dyslipidemia

Previous studies showed that treatment with hypolipidemic drugs, such as statins, was more frequent in COPD patients [21]. To exclude the impact of hypolipidemic treatment on serum lipid, 3 studies enrolling patients with stable COPD who were not receiving treatment for dyslipidemia were included in the subgroup analysis. No significant differences were found in the HDL (MD = -4.33, 95% CI [-8.76, 0.10], P = 0.06) (Fig. 3a), LDL (MD = -3.05, 95% CI [-9.50, 3.39], P = 0.35) (Fig. 3b) and TC (MD = -1.51, 95% CI [-10.40, 7.38], P = 0.74) (Fig. 3c) levels of the 2 groups. However, serum TG level was significantly higher in subjects with stable COPD than in control subjects (MD = 16.35, 95% CI [5.90, 26.80], P = 0.002) (Fig. 3d).

### Sensitivity analysis

Sensitivity analysis was performed by sequential removal (statistics of study remove) of individual studies. There was no significant change in the overall outcomes after removing any of the studies, indicating that the meta-analysis had good reliability and that the data was not overly influenced by any given study.

### Risk of bias assessment

**Fig. 4:**
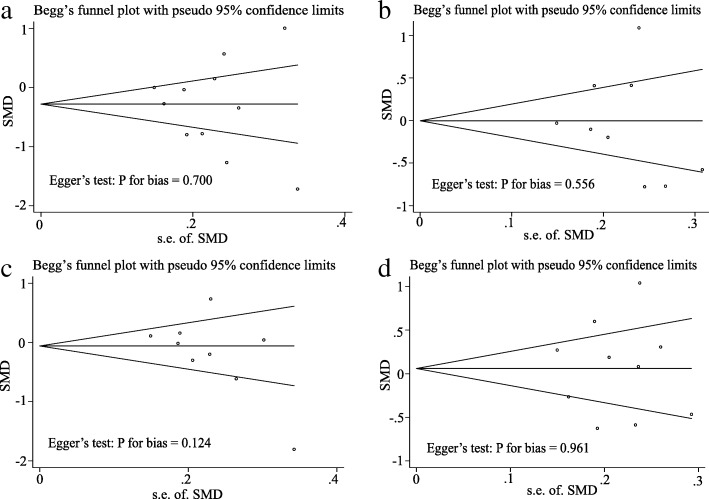
Begg’s funnel plot analysis and Egger’s test to detect publication bias. **a** Begg’s funnel plot analysis and Egger’s test for HDL. **b** Begg’s funnel plot analysis and Egger’s test for LDL. **c** Begg’s funnel plot analysis and Egger’s test for TC. **d** Begg’s funnel plot analysis and Egger’s test for TG

Begg’s and Egger’s test was used to detect potential publication bias. The Funnel plots’ shape did not reveal obvious evidence of asymmetry, and all the P value of Egger’s test was more than 0.05 (Fig. 4a, b, c, and d). Thus, the above results suggest that publication bias was not evident in this meta-analysis. As only 3 studies were included in the subgroup analysis, publication bias could not be assessed.

## Discussion

Several studies have indicated that the prevalence of metabolic syndrome is significantly higher in COPD patients compared to healthy controls. Dyslipidemia is an important component of the metabolic syndrome and is included in definitions of the metabolic syndrome published by different international committees [24]. Several studies have evaluated the relationship between COPD and blood lipid profiles, but the results remain controversial [13, 14, 17, 19]. In this meta-analysis, we compared the serum levels of HDL, LDL, TC, and TG between the COPD and healthy control groups.

Previous conclusions of numerous studies on the relationship between COPD and blood lipid profiles remain conflicting and contradictory. The reported associations of blood lipid profiles with COPD have been reported as positive, negative, or no association. Sibel et al. found that serum HDL level was significantly lower, while TG level was significantly higher in subjects with stable COPD than in control subjects [13]. Jiayu et al. reported that there was no difference between serum TG, TC, and LDL levels of COPD patients and control individuals [14]. On the basis of 11 studies, this meta-analysis indicated that HDL, LDL, TC, and TG showed similar levels between COPD patients and control individuals. The higher proportion of COPD patients being on oral hypolipemic agents, such as stains, compared to control participants might contribute to this contradiction [19]. Therefore, 3 studies enrolling patients who were not receiving treatment for dyslipidemia were included in the subgroup analysis. The results of the subgroup analysis revealed that HDL, LDL, and TC levels were similar between COPD patients and controls, while TG level was significantly higher in subjects with stable COPD. However, as only 3 studies were included in our subgroup analysis, these results should be interpreted with caution.

Other potential interfering factors such as gender, lifestyle, BMI, disease severity, and smoking may also impact levels of the blood lipids and hence, their association with COPD. Breyer et al. reported that metabolic syndrome is more prevalent in overweight to obese patients with COPD compared to BMI matched healthy subjects. No difference in the frequency of metabolic syndrome was observed in low to normal weight patients and healthy subjects [25]. The severity of COPD may also impact serum lipid levels. Ummugulsum Can et al. investigated the association between disease severity and serum lipid levels in different Global Initiative for Chronic Obstructive Lung Disease (GOLD) stages of patients and control individuals. They found that patients with GOLD Stages III and IV had significantly lower HDL levels than control individuals. However, they saw no difference between patients with GOLD Stages II and healthy controls with respect to HDL levels [14]. As there are limited included studies and insufficient participants in our research, we didn’t perform subgroup analysis according to BMI and disease severity. More studies with larger sample size and multiple subgroups are needed to further explore this association.

The following mechanisms may be responsible for the predisposition of patients with COPD to develop dyslipidemia. Firstly, systemic inflammation plays an important role in both COPD and dyslipidemia [26–28]. Inflammation itself is associated with decreased serum HDL and increased TG levels [29]. Studies showed that inflammatory cytokines could promote disruption of lipid metabolism. For example, an inverse correlation exists between serum HDL and IL-6 levels. Conversely, increased IL-10 concentration is associated with raised plasma HDL [29]. Secondly, COPD patients are notably physical inactive in daily life, which could increase their risk of dyslipidemia [10, 11, 30]. Thirdly, corticosteroid are widely used in patients with COPD, especially in those with acute exacerbations. However, patients with high corticosteroid levels may suffer from a variety of diseases, including obesity and dyslipidemia. For example, one study reported that 7 weeks of dexamethasone treatment facilitated diet-induced dyslipidemia [31, 32]. Another population based study showed that low-dose short-term corticosteroids markedly affect plasma lipid levels [33]. However, the impact of corticosteroid use on lipid levels in patients with COPD is still unknown and requires further well-designed studies. Fourthly, cigarette smoking, as well as oxidative stress are possible mechanisms responsible for the development of dyslipidemia in COPD patients [12, 34].

There are several limitations in the present study that should be specified. First, the analysis did not stratify by disease severity, gender, BMI, and smoking. Second, the number of articles included in this research is relatively small. More well-designed studies with larger sample sizes are required to confirm and explore these results.

## Conclusions

In summary, this meta-analysis found no significant relationship between COPD and the serum levels of HDL, LDL, TC and TG. However, subgroup analysis enrolling patients who were not receiving treatment for dyslipidemia showed that TG levels were higher in patients with COPD than in healthy individuals. These findings suggest that physicians should screen COPD patients for elevated TG levels to reduce the risk of cardiovascular morbidity and mortality. Our findings must be interpreted with caution because of the small sample size and limitations of the study. More well-designed studies with larger sample size and multiple subgroups are needed to further explore this association.

## Abbreviations

BMI: Body mass index
CHD: Coronary heart disease
CI: Confidence intervals
COPD: Chronic obstructive pulmonary disease
GOLD: Global Initiative for Chronic Obstructive Lung Disease
HDL: High-density lipoprotein cholesterol
LDL: Low-density lipoprotein cholesterol
MD: Mean differences
TC: Total cholesterol
TG: Triglyceride
